# Personal

**Published:** 1915-09

**Authors:** 


### PERSONAL.

---

**Announcement of removal, travel, and other matters of interest are requested. Please report errors in the listing of any physician in the State and other directories, that we may co-operate with the State Society in securing a correct list.**

---

Dr. Charles R. Cullinane of Buffalo returned August 3 from a motor trip through the White Mountains.

---

Dr. George L. Brown of Buffalo returned July 20 from a two months' trip to the Pacific Coast.

---

Dr. B. H. Grove of Buffalo returned from an auto tour through New England, August 1.

Dr. Roland O. Meisenbach of Buffalo gave a clinic at Los Angeles, by invitation, after the meeting of the A. M. A.

Dr. C. B. Kibler of Corry, Pa., made a tour of New York and New England in August.

The touring car of Dr. E. E. Haley of Buffalo, was stolen on the night of August 18 but was found, undamaged, the next morning.

